# Loss of ncm^5^ and mcm^5^ wobble uridine side chains results in an altered metabolic profile

**DOI:** 10.1007/s11306-016-1120-8

**Published:** 2016-09-27

**Authors:** Tony Karlsborn, A. K. M. Firoj Mahmud, Hasan Tükenmez, Anders S. Byström

**Affiliations:** Department of Molecular Biology, Umeå University, 901 87 Umeå, Sweden

**Keywords:** Elongator complex, tRNA wobble uridine modifications, Translation, *ELP3*, Metabolomics, Metabolic profiling

## Abstract

**Introduction:**

The Elongator complex, comprising six subunits (Elp1p-Elp6p), is required for formation of 5-carbamoylmethyl (ncm^5^) and 5-methoxycarbonylmethyl (mcm^5^) side chains on wobble uridines in 11 out of 42 tRNA species in *Saccharomyces cerevisiae*. Loss of these side chains reduces the efficiency of tRNA decoding during translation, resulting in pleiotropic phenotypes. Overexpression of hypomodified ${\text {tRNA}_{{\rm s^{2} {\rm UUU}}}^{{\rm Lys}} , {\rm tRNA}_{{\rm s^{2} {\rm UUG}}}^{{\rm Gln }} \;{\rm and}\;{\rm tRNA}_{{\rm s^{2} {\rm UUC}}}^{{\rm Glu}}}$, which in wild-type strains are modified with mcm^5^s^2^U, partially suppress phenotypes of an *elp3Δ* strain.

**Objectives:**

To identify metabolic alterations in an *elp3Δ* strain and elucidate whether these metabolic alterations are suppressed by overexpression of hypomodified ${\text {tRNA}_{{\rm s^{2} {\rm UUU}}}^{{\rm Lys}} , {\rm tRNA}_{{\rm s^{2} {\rm UUG}}}^{{\rm Gln }} \;{\rm and}\;{\rm tRNA}_{{\rm s^{2} {\rm UUC}}}^{{\rm Glu}}}$.

**Method:**

Metabolic profiles were obtained using untargeted GC-TOF-MS of a temperature-sensitive *elp3Δ* strain carrying either an empty low-copy vector, an empty high-copy vector, a low-copy vector harboring the wild-type *ELP3* gene, or a high-copy vector overexpressing ${\text {tRNA}_{{\rm s^{2} {\rm UUU}}}^{{\rm Lys}} , {\rm tRNA}_{{\rm s^{2} {\rm UUG}}}^{{\rm Gln }} \;{\rm and}\;{\rm tRNA}_{{\rm s^{2} {\rm UUC}}}^{{\rm Glu}}}$. The temperature sensitive *elp3Δ* strain derivatives were cultivated at permissive (30 °C) or semi-permissive (34 °C) growth conditions.

**Results:**

Culturing an *elp3Δ* strain at 30 or 34 °C resulted in altered metabolism of 36 and 46 %, respectively, of all metabolites detected when compared to an *elp3Δ* strain carrying the wild-type *ELP3* gene. Overexpression of hypomodified ${\text {tRNA}_{{\rm s^{2} {\rm UUU}}}^{{\rm Lys}} , {\rm tRNA}_{{\rm s^{2} {\rm UUG}}}^{{\rm Gln }} \;{\rm and}\;{\rm tRNA}_{{\rm s^{2} {\rm UUC}}}^{{\rm Glu}}}$ suppressed a subset of the metabolic alterations observed in the *elp3Δ* strain.

**Conclusion:**

Our results suggest that the presence of ncm^5^- and mcm^5^-side chains on wobble uridines in tRNA are important for metabolic homeostasis.

**Electronic supplementary material:**

The online version of this article (doi:10.1007/s11306-016-1120-8) contains supplementary material, which is available to authorized users.

## Introduction

In eukaryotes, the Elongator complex is highly-conserved and comprises six subunits (Elp1p–Elp6p) (Otero et al. 1999; Y. Li et al. 2001; Krogan and Greenblatt 2001; Winkler et al. 2001; Hawkes et al. 2002; Nelissen et al. 2005). The complex is required for formation of 5-carbamoylmethyluridine (ncm^5^U), 5-methoxycarbonylmethyluridine (mcm^5^U) and 5-methoxycarbonylmethyl-2-thiouridine (mcm^5^s^2^U) modifications at wobble positions in tRNAs (Huang et al. 2005; Esberg et al. 2006; Chen et al. 2009; Lin et al. 2013; Mehlgarten et al. 2010; Karlsborn et al. 2014a). In *Saccharomyces* cerevisiae, loss-of-function mutations in any gene encoding an Elongator complex subunit gives rise to a multitude of phenotypes linked to several different cellular processes (Otero et al. 1999; Wittschieben et al. 1999; Winkler et al. 2002; Rahl et al. 2005; Tigano et al. 2015; Nedialkova and Leidel 2015; Frohloff et al. 2001; Chen et al. 2011; Q. Li et al. 2009). Phenotypes observed in yeast Elongator mutants, except the tRNA modification defect, are suppressed by overexpression of ${\text {tRNA}_{{\rm s^{2} {\rm UUU}}}^{{\rm Lys}} \; {\rm and} \; {\rm tRNA}_{{\rm s^{2} {\rm UUG}}}^{{\rm Gln }}}$, which have the mcm^5^s^2^U modification in wild-type yeast. This discovery highlighted the importance of Elongator-complex-dependent tRNA modifications in translation (Esberg et al. 2006).

In an *elp3Δ* mutant enrichment of lysine-AAA codons in mRNAs decoded by ${\text{tRNA}}_{{{\text{mcm}}^{{\text{5}}} {\text{s}}^{{\text{2}}} {\text{UUU}}}}^{{{\text{Lys}}}}$ having the Elongator complex dependent wobble uridine modification mcm^5^s^2^U result in reduced protein expression (Bauer et al. 2012). Replacing these lysine-AAA codons with the near-cognate G-ending AAG codon, decoded by a tRNA isoacceptor not requiring the Elongator complex dependent wobble uridine modification improved protein expression from the codon altered gene (Fernandez-Vazquez et al. 2013; Bauer et al. 2012). Moreover, ribosomal profiling studies performed with Elongator mutants revealed ribosomal pausing at the lysine-AAA and glutamine-CAA codons (Nedialkova and Leidel 2015; Zinshteyn and Gilbert 2013) and possibly the glutamic acid-GAA codons (Zinshteyn and Gilbert 2013). These results support the previous suggestion that the presence of the mcm^5^s^2^U modification in ${\text{tRNA}}_{{{\text{mcm}}^{{\text{5}}} {\text{s}}^{{\text{2}}} {\text{UUU}}}}^{{{\text{Lys}}}} \; {\rm and} \; {\text{tRNA}}_{{{\text{mcm}}^{{\text{5}}} {\text{s}}^{{\text{2}}} {\text{UUG}}}}^{{{\text{Gln}}}}$ enhance translational efficiency (Esberg et al. 2006), probably because of improved codon-anticodon interactions (Johansson et al. 2008; Durant et al. 2005; Bauer et al. 2012; Vendeix et al. 2012; Rezgui et al. 2013; Tükenmez et al. 2015). However, whether the phenotypes are caused by global reduction of protein expression or altered protein expression from specific mRNAs, leading to downstream effects, is unknown. Moreover, whether the loss of modified wobble uridines causes metabolic alterations is yet to be determined.

Our study demonstrates that a large number of metabolites within an *elp3Δ* strain undergoes perturbed metabolism. Furthermore, the range of metabolites with altered levels expanded with growth of the *elp3Δ* strain at 34 °C; this is probably an effect of the temperature sensitivity phenotype of the *elp3Δ* mutant. Our study also shows that elevated levels of ${\text {tRNA}_{{\rm s^{2} {\rm UUU}}}^{{\rm Lys}} , {\rm tRNA}_{{\rm s^{2} {\rm UUG}}}^{{\rm Gln }} \;{\rm and}\;{\rm tRNA}_{{\rm s^{2} {\rm UUC}}}^{{\rm Glu}}}$ in the *elp3Δ* strain suppress some, but not all metabolic alterations.

## Methods

### Yeast strains, media, and genetic procedures

Yeast strains and plasmids used in this study are found in Online Resource 1 (Christianson et al. 1992; Lu et al. 2005; Sikorski and Hieter 1989). Genetic procedures, media, and yeast transformation have been described previously (Burke et al. 2000; Gietz and Schiestl 2007). An *elp3* null mutant was generated by linear transformation of the diploid strain UMY2016/UMY2026 with an *elp3::kanMX4* fragment (fragment amplified by polymerase chain reaction (PCR)) generated from the *elp3Δ* strain in the yeast deletion collection (Open Biosystems). Transformants were selected on YEPD plates containing 200 µg/ml of G418. The heterozygous diploid generated was sporulated and tetrad dissection generated haploids UMY4238 and UMY4239. Insertion of the *elp3::KanMX4* cassette in UMY4239 was verified by PCR and high performance liquid chromatography (HPLC) analysis was used to determine the status of the wobble uridine nucleosides: ncm^5^U, mcm^5^U and mcm^5^s^2^U in yeast tRNA (Huang et al. 2005).

### Cell sampling and metabolite extraction using untargeted GC-TOF-MS metabolomics

Strains UMY4239 (*elp3::KanMX4*) and UMY4238 were transformed with one of the following plasmids: empty pRS315, pRS315 containing the wild-type *ELP3* gene, pRS425 or pRS425 containing the tRNA genes *tK(UUU)*, *tQ(UUG)* and *tE(UUC)*. Three replicates of each strain derivative were cultivated in synthetic defined media at either 30 or 34 °C until cell density was ~0.5 OD_600_ units. At ~0.5 OD_600_ units, cells amounting to 1 OD unit were harvested in triplicate from each biological replicate by centrifugation at 0 °C. The supernatant was discarded and the cells were washed with 2 ml of ice-cold phosphate buffered saline (PBS) then centrifuged again at 0 °C. The supernatant was discarded and pellets were suspended in a 90:10 mixture of Methanol and MilliQ (MQ) water which was pre-chilled on dry ice. Suspended pellets were stored at −80 °C until metabolite extraction.

Metabolites were extracted by grinding the pellets with glass beads for 3 min at 30 Hz followed by centrifugation at 14,000 RPM for 10 min. A 200 µL aliquot of the supernatant was transferred to a GC-vial and evaporated using a SpeedVac. Derivatization of the metabolic extract was performed using 30 µL of methoxyamine (16 h at room temperature). The extract was then trimethylsilylated by adding 30 µL of *N*-methyl-*N*-trimethylsilyltrifluoroacetamide (MSTFA) to the vial and incubating for 1 h at 25 °C. Subsequently, 30 µL of heptane containing 15 ng/µL methyl stearate was added to the vial.

Samples were analysed using combined gas chromatography with time-of-flight mass spectrometry (GC/TOFMS). For retention indices, an *n*-alkane series (C8–C40) was included in the analysis (Schauer et al. 2005). A 1 μL volume of derivatized sample was injected into a split/splitless injector, in splitless mode, on an Agilent CTC PAL Systems Autosampler with a 10 μL syringe (Agilent Technologies, Atlanta, GA, USA). The autosampler injected samples into an Agilent Technologies 7890A GC System (Agilent Technologies, Atlanta, GA, USA). The Agilent Technologies 7890A GC System was equipped with a 30 m × 0.250 mm-diameter fused, silica capillary column with a bonded 0.25 μm Durabond DB-5MSUI stationary phase (part no: 122-5222UI, Agilent J&W GC columns). The injector temperature was set to 260 °C, front inlet septum purge flow set to 3 mL min^−1^, and gas flow rate through the column set to 1 mL min^−1^. Column temperature was held at 70 °C for 2 min, then increased by 20 °C min^−1^ to 320 °C, and held for 8 min. The column effluent was led into the ion source of a Pegasus HT GC-TOF-MS (LECO Corp., St Joseph, MI, USA). The transfer line and ion source temperatures were 270 and 200 °C, respectively. Detector voltage was set to 1650 V. Ions were generated by a −70 V electron beam at an ionization current of 2.0 mA, and 20 spectra s^−1^ were recorded in the mass range 50–800 m/z. The acceleration voltage was turned on after a solvent delay of 270 s.

### Data processing of samples subjected to GC-TOF-MS

Unprocessed MS files from GC/TOF–MS analysis were exported in NetCDF format to MATLAB software R2013a (Mathworks, Natick, MA). All data pretreatment procedures, including baseline correction, chromatogram alignment, time-window setting and multivariate curve resolution (MCR) (Jonsson et al. 2005) were performed in MATLAB using custom scripts. Peak detection against mass spectra libraries (targeted data processing) was performed with an in-house script. Metabolites were identified by using NIST MS Search 2.0 software to compare the mass spectra of all detected compounds with spectra in: the NIST library 2.0, the in-house mass spectra library established by Swedish Metabolomics Centre, and the mass spectra library maintained by the Max Planck Institute in Golm (http://csbdb.mpimp-golm.mpg.de/csbdb/gmd/gmd.html).

A retention index comparison was performed, with a retention index deviation < ± 10 (in addition to a high spectral match) resulting in a positive ID. Generated peaks were analysed in tandem using the spectral database found at www.massbank.jp. The data was normalized using all 11 internal standards (eluting over the whole chromatographic time range). A principal component analysis (PCA), using peak areas for the internal standards, was conducted and the T-score value for each sample was used to normalize the resolved data by dividing the peak areas of each sample with its corresponding score value. Multivariate analysis was performed with SIMCA-P + 13 software (Umetrics AB, Umeå, Sweden). Data from the analysis and peak-spectra is available as (Online Resource 12–13).

### Data analysis

Data was preprocessed for an integrity check and transformed into the binary logarithm [base of 2; log(2)] for downstream analysis (Stacklies et al. 2007). Extreme outliers were replaced by the median of the data within biological replicates, and data was subjected to pareto scaling (Dieterle et al. 2006). Heatmaps were generated using the heatmap.2 function in the gplots package in R software with data transformed into the common logarithm [base of 10; log(10)] using averages of metabolite levels.

PCA is an unsupervised method for finding the directions that best explain the variance in a data set (X) without referring to classification labels (Y). PCA was performed using the prcomp syntax in R (William N. Venables 2002) or SIMCA, version 14.0.0.1359 (Umetrics AB, Umeå, Sweden).

Partial least-squares discriminant analysis (PLS-DA) is a supervised method that uses multivariate regression techniques to extract information that can predict classification (Y) via linear combination of original variables (X). PLS-DAs were performed on log(2)-transformed metabolite concentrations that had been centered according to the means using SIMCA, version 14.0.0.1359 (Umetrics AB, Umeå, Sweden); unit-variance scaling was applied as previously described (Slupsky et al. 2007).

A permutation test was performed (20 permutations) to assess the significance of classification, and prediction accuracy was determined (Max Kuhn. Contributions from Jed Wing and Steve Weston and Andre Williams. caret: Classification And REgression Training, 2008, R package version 3.45) (Bijlsma et al. 2006). Variable Importance in Projection (VIP) in PLS-DA is a weighted sum of the squares of the PLS loadings that accounts for the amount of explained Y-variation in each dimension for each component (Max Kuhn. Contributions from Jed Wing and Steve Weston and Andre Williams. caret: Classification And Regression Training, 2008).

## Results

### Loss of ncm^5^U, mcm^5^U and mcm^5^s^2^U wobble uridine nucleosides in tRNA result in an altered metabolic profile

**Fig. 1:**
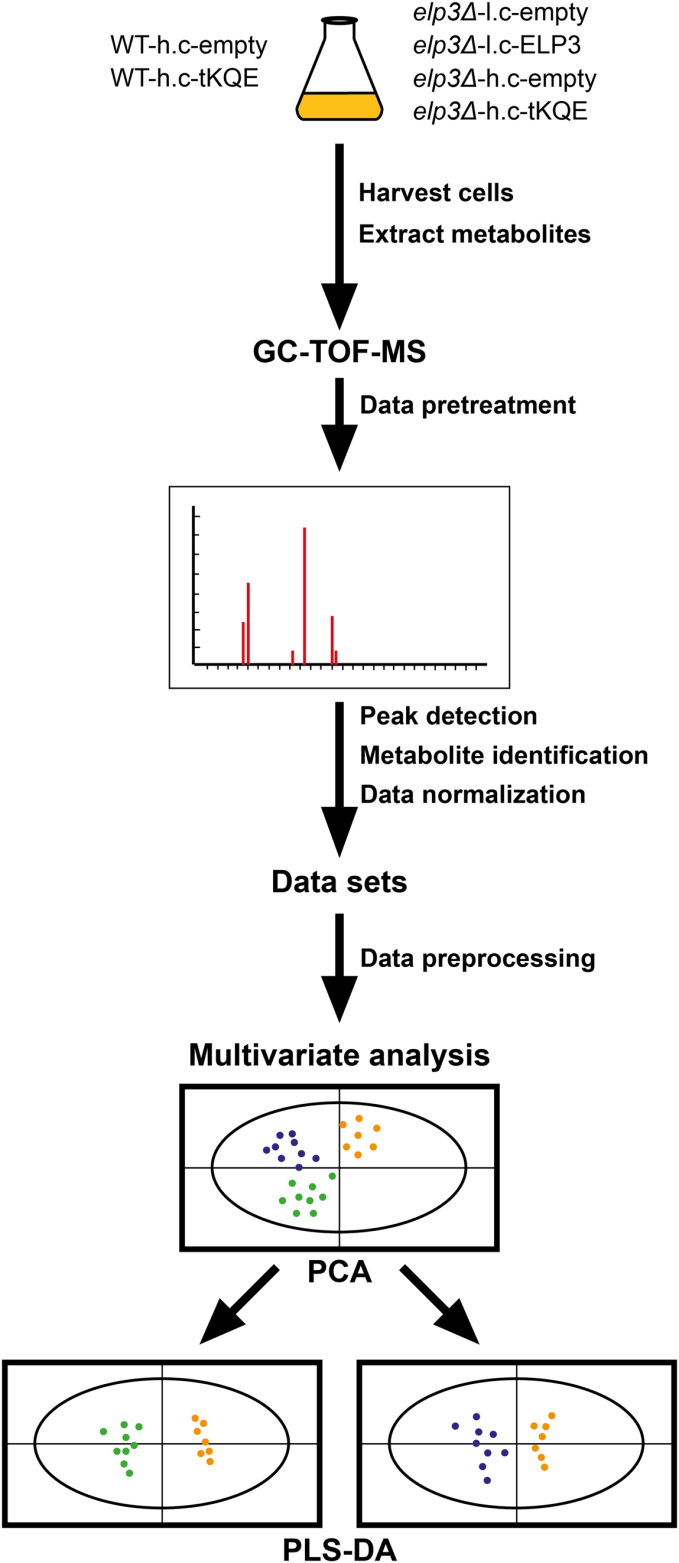
Overview of the pipeline for metabolic profiling of wild-type and *elp3Δ* strains carrying indicated plasmids. The UMY4238 and UMY4239 yeast strains contained either: an empty low-copy pRS315 vector (*elp3Δ*-l.c.-empty); a pRS315 vector containing the wild-type *ELP3* gene (*elp3Δ*-l.c.-ELP3); an empty high copy pRS425 vector (*elp3Δ*-h.c.-empty, WT-h.c.-empty); or a high copy pRS425 vector carrying the tRNA genes *tK(UUU)*, *tQ(UUG)* and *tE(UUC)* (*elp3Δ*-h.c.-tKQE, WT-h.c.-tKQE). Yeast strains were cultivated logarithmically to an OD_600_ value of ~0.5 at 30 or 34 °C and harvested. Metabolites were extracted and then quantified using GC-TOF-MS. Metabolite data was analyzed using multivariate analysis (PCA, PLS-DA) which separated the metabolites according to different classes representing the *elp3Δ* strains containing various plasmid constructs

We subjected an *elp3Δ* strain carrying either an empty low copy *LEU2* vector (*elp3Δ*-l.c.-empty) or the same vector containing the wild-type *ELP3* gene (*elp3Δ*-l.c.-*ELP3*) to metabolic profiling using non-targeted GC-TOF-MS (Fig. 1). This metabolic profiling was conducted to investigate whether loss of the ncm^5^U, mcm^5^U and mcm^5^s^2^U wobble uridine nucleosides in yeast tRNA causes metabolic alterations. We also included samples of the wild-type strain carrying an empty high-copy *LEU2* vector (WT-h.c.-empty) to investigate whether metabolism of the *elp3Δ*-l.c.-*ELP3* strain represents that of the wild-type strain.

**Fig. 2:**
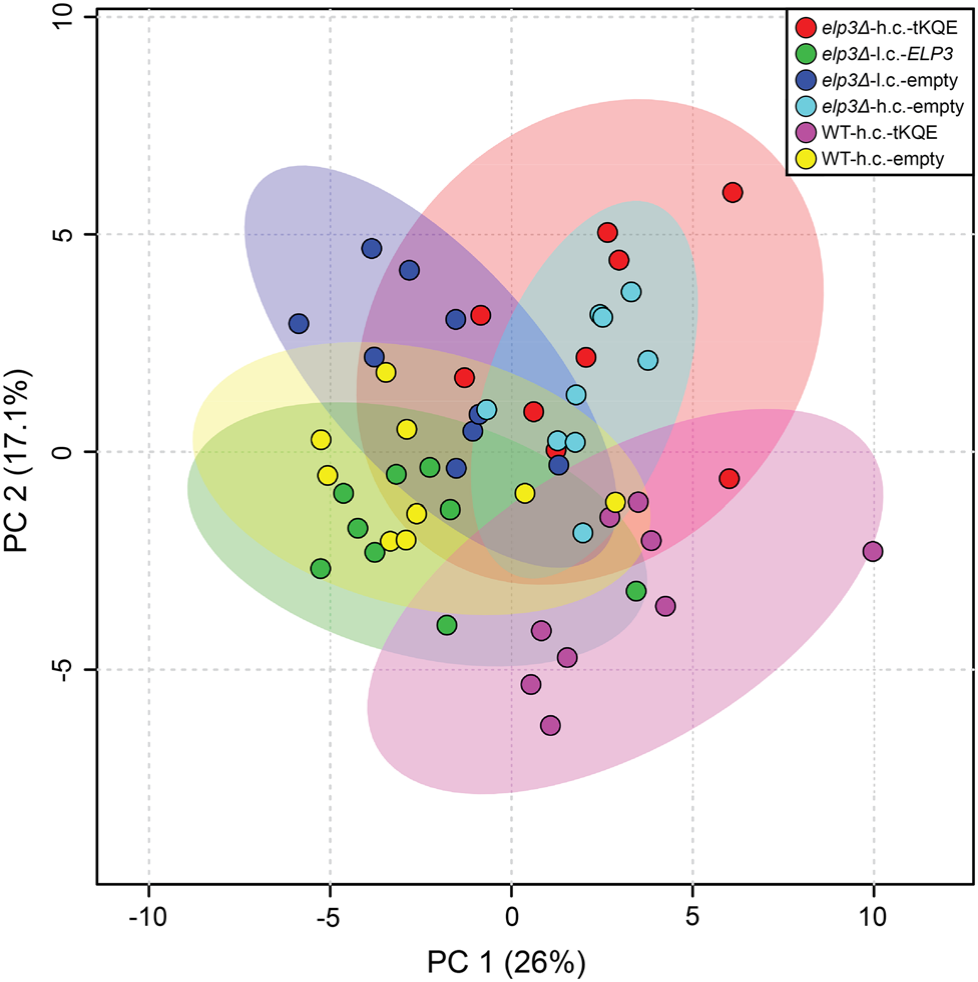
Metabolic variation of the wild-type and *elp3Δ* strain containing indicated plasmids. The UMY4238 and UMY4239 yeast strains containing either: an empty low-copy pRS315 vector (*elp3Δ*-l.c.-empty); a pRS315 vector containing the wild-type *ELP3* gene (*elp3Δ*-l.c.-ELP3); an empty high copy pRS425 vector (*elp3Δ*-h.c.-empty, WT-h.c.-empty); or a high copy pRS425 vector carrying the tRNA genes *tK(UUU)*, *tQ(UUG)* and *tE(UUC)* (*elp3Δ*-h.c.-tKQE, WT-h.c.-tKQE) were grown logarithmically to an OD_600_ of ~0.5 at 30 °C and harvested. Metabolites were extracted and then quantified using GC-TOF-MS. Each* dot* in the PCA analysis represents a technical replicate from three different biological replicates

Since *elp3Δ* strains are temperature sensitive (Ts), we cultured strains to ~0.5 OD_600_ units under permissive (30 °C) and semi-permissive (34 °C) growth conditions, as metabolic changes may be more pronounced at elevated temperatures. From our metabolite extracts, 111 metabolites could be measured using GC-TOF-MS, 41 of which could be identified while the remaining were unidentified. We performed a PCA of all the strains used and all 111 metabolites detected in this study to get an overview of metabolism in the strains. The PCA results showed that the *elp3Δ*-l.c.-*ELP3* strain clusters with the WT-h.c.-empty strain, indicating that metabolism in these strains is similar (Fig. 2).

**Fig. 3:**
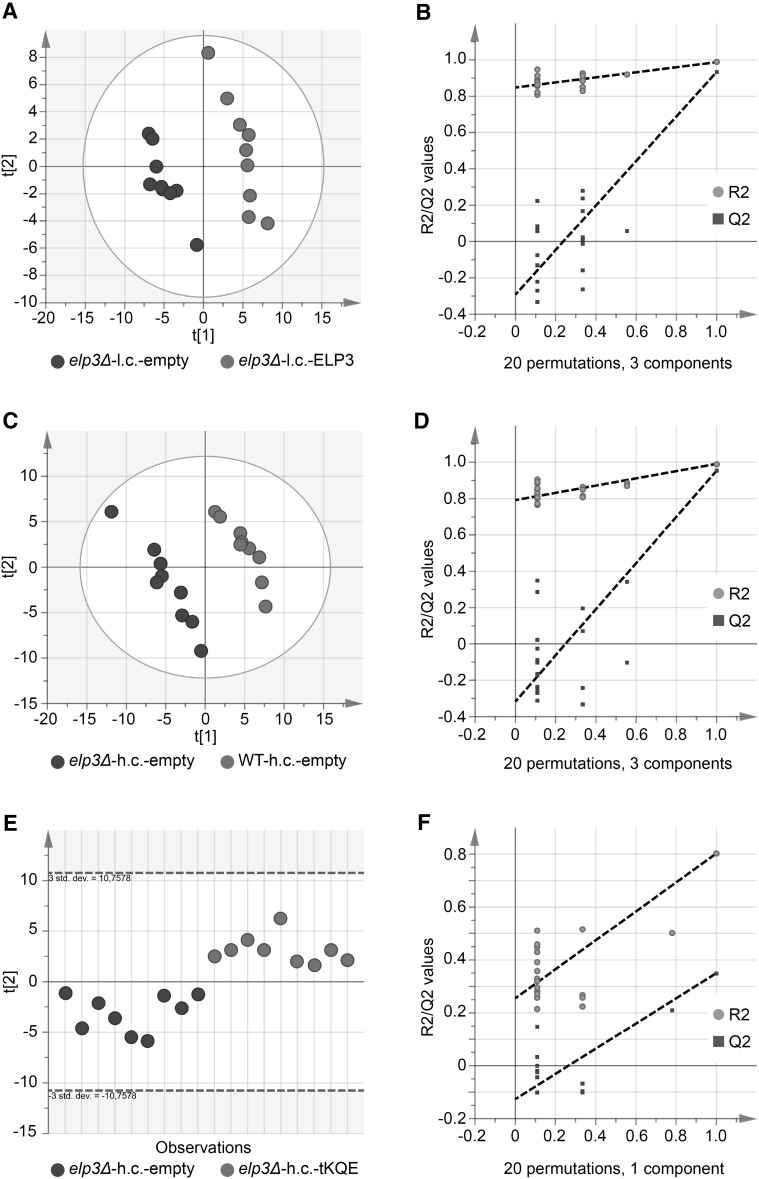
Score plots summarizing the PLS-DA modelling of strains grown at 30 °C. **a** PLS-DA score plot when modelling the *elp3Δ* strain with an empty low copy pRS315 vector (*elp3Δ*-l.c.-empty) against the *elp3Δ* strain containing the wild-type *ELP3* gene on a pRS315 vector (*elp3Δ*-l.c.-ELP3). **b** Random permutation (20 randomizations) test-validation plot of PLS-DA model in (**a**). **c** PLS-DA score plot when modelling the *elp3Δ* strain with an empty high copy pRS425 vector (*elp3Δ*-h.c.-empty) against the wild-type strain with an empty pRS425 vector (WT-h.c.-empty). **d** Random permutation (20 randomizations) test-validation plot of PLS-DA model in (**c**). **e** PLS-DA score plot when modelling the *elp3Δ* strain with an empty high copy pRS425 vector (*elp3Δ*-h.c.-empty) against the *elp3Δ* strain containing a pRS425 vector carrying the tRNA genes *tK(UUU)*, *tQ(UUG)* and *tE(UUC)* (*elp3Δ*-h.c.-tKQE). **f** Random permutation (20 randomizations) test-validation plot of PLS-DA model in (**e**). The PLS-DA model in **e** only has one valid component and is therefore portrayed by one vector. Each *dot* in **a**, **c** and **e** represents a technical replicate from three different biological replicates

**Fig. 4:**
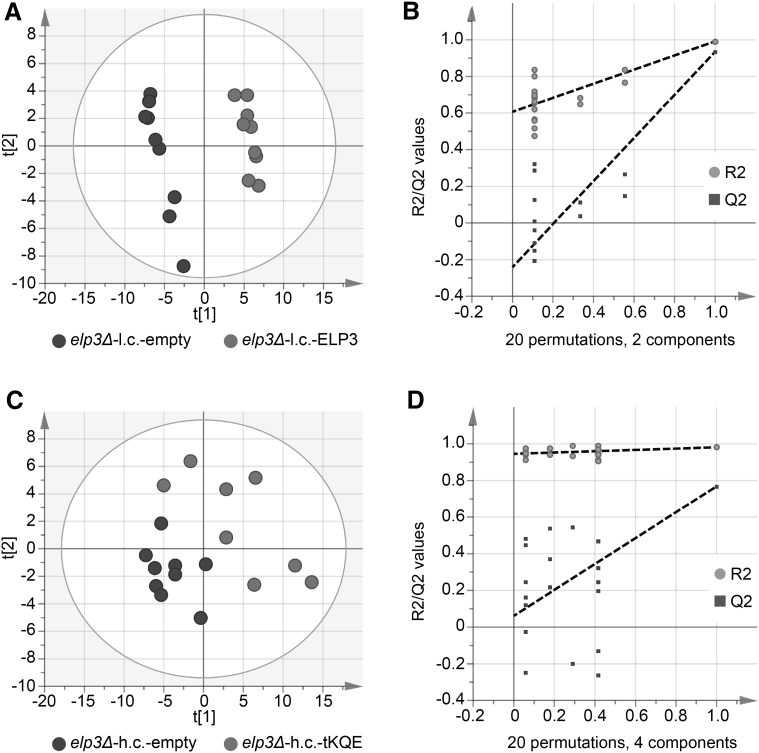
Score plots summarizing the PLS-DA modelling of strains grown at 34 °C. **a** PLS-DA score plot when modelling the *elp3Δ* strain with an empty low copy pRS315 vector (*elp3Δ*-l.c.-empty) against the *elp3Δ* strain containing the wild-type *ELP3* gene on a pRS315 vector (*elp3Δ*-l.c.-ELP3). **b** Random permutation (20 randomizations) test-validation plot of PLS-DA model in **a**. **c** PLS-DA score plot when modelling the *elp3Δ* strain with an empty high copy pRS425 vector (*elp3Δ*-h.c.-empty) against the *elp3Δ* strain containing a pRS425 vector carrying the tRNA genes *tK(UUU)*, *tQ(UUG)* and *tE(UUC)* (*elp3Δ*-h.c.-tKQE). **d** Random permutation (20 randomizations) test-validation plot of PLS-DA model in (**c**). Each *dot* in **a**, **c** and **e** represents a technical replicate from three different biological replicates

We looked for alterations in levels of specific metabolites in the *elp3Δ* strain using partial least squares regression discriminant analysis (PLS-DA) (Figs. 3a–f, 4a–d). Metabolites with a variable importance for the projection (VIP) below one were excluded (Chong and Jun 2005). Briefly, PLS-DA allows analysis of large sample sets structured in the form of classes. The classes are separated according to a comparison between all variables within one class and all variables within another class, and subsequent prediction of the variables that account for the class separation. Variables that are good predictors for separating one class from another have a high VIP score, while variables with a low VIP score do not contribute to class separation.

**Fig. 5:**
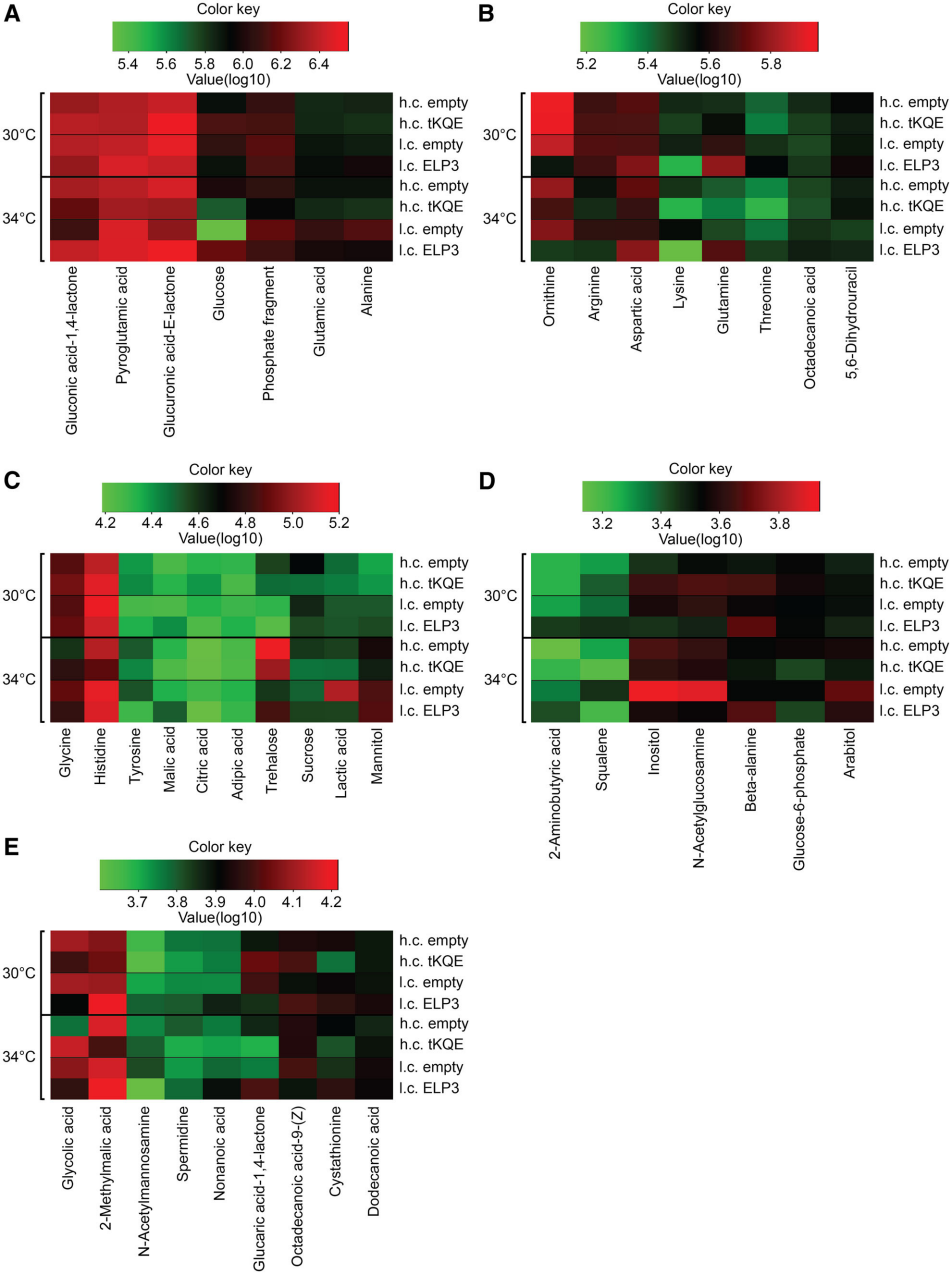
Metabolic alterations upon loss of ncm^5^U, mcm^5^U and mcm^5^s^2^U wobble uridine nucleosides in tRNA. The UMY4239 *elp3Δ* strains contained either: an empty pRS315 low copy vector (l.c. empty); a pRS315 vector carrying the wild-type *ELP3* gene (l.c. ELP3); an empty pRS425 high copy vector (h.c. empty); or a pRS425 high copy vector carrying the tRNA genes *tK(UUU)*; *tQ(UUG)* and *tE(UUC)* (h.c. tKQE). These yeast strains were grown logarithmically to ~0.5 OD_600_ at 30 or 34 °C and harvested. Metabolites were extracted and then quantified using GC-TOF-MS. Metabolites were hierarchically organized into five clusters as represented by **a**–**e** based on metabolite abundance (see Online Resource 2). *Red* signifies metabolite enrichment and *green* signifies metabolite reduction

A comparison of the metabolic profiles of only identified metabolites showed that permissive growth (30 °C) of the *elp3Δ*-l.c.-empty strain resulted in elevated levels of primarily Ornithine, Lysine and *N*-Acetylglucosamine, and reduced levels of Glutamine, Beta-alanine, Malic acid, Aspartic acid, Pyroglutamic acid, Alanine, Threonine, 2-Aminobutyric acid, 5,6-Dihydrouracil and Tyrosine when compared to the *elp3Δ*-l.c.-*ELP3* strain (Fig. 5a–e, Online Resource 2–3). Semi-permissive growth (34 °C) of the *elp3Δ*-l.c.-empty strain also resulted in elevated levels of Ornithine, Lysine and N-Acetylglucosamine when compared to the *elp3Δ*-l.c.-*ELP3* strain (Fig. 5a–e, Online Resource 2–3). We also observed that the *elp3Δ*-l.c.-empty strain accumulated eight additional metabolites with known identity at 34 °C (Online Resource 4), indicating more pronounced metabolic changes with growth at elevated temperatures.

Semi-permissive (34 °C) growth of the *elp3Δ*-l.c.-empty strain showed reduced levels of Beta-alanine and Glutamine, as observed at permissive growth (30 °C) (Fig. 5a–e, Online Resource 2–4). In addition, semi-permissive growth of the *elp3Δ*-l.c.-empty strain showed reduced levels of Malic acid, Aspartic acid and Threonine. Surprisingly, we did not observe significantly reduced levels of Pyroglutamic acid, Alanine, 2-Aminobutyric acid, 5,6-Dihydrouracil and Tyrosine after semi-permissive growth, as when the *elp3Δ*-l.c.-empty strain grew at 30 °C. Instead, semi-permissive growth of this strain resulted in reduced levels of Gluconic acid 1,4-lactone, Glucose, Trehalose, Octadecanoic acid and Glucuronic acid-E-lactone.

Moreover, we observed that several unidentified metabolites had a VIP score above one, indicating altered metabolism of these metabolites in the *elp3Δ*-l.c.-empty strain compared to the *elp3Δ*-l.c.-*ELP3* strain at both 30 and 34 °C (Online Resource 5–6). The amounts of identified and unidentified metabolites with altered levels in the *elp3Δ*-l.c.-empty strain and the *elp3Δ*-l.c.-*ELP3* strain were higher when these strains grew at 34 °C (Online Resource 5–6). Our results show that semi-permissive growth of the *elp3Δ*-l.c.-empty strain resulted in more metabolic alterations than permissive growth. More metabolites started to increase or decrease at 34 °C in the *elp3Δ*-l.c.-empty strain while levels of these metabolites were more or less constant at both 30 and 34 °C in the *elp3Δ*-l.c.-*ELP3* strain.

### Elevated levels of hypomodified ${\text {tRNA}_{{\rm s^{2} {\rm UUU}}}^{{\rm Lys}} , {\rm tRNA}_{{\rm s^{2} {\rm UUG}}}^{{\rm Gln }} \;{\rm and}\;{\rm tRNA}_{{\rm s^{2} {\rm UUC}}}^{{\rm Glu}}}$ partially suppress certain metabolic alterations

All phenotypes tested in yeast Elongator mutants to date have been partially suppressed by elevated levels of various combinations of hypomodified ${\text {tRNA}_{{\rm s^{2} {\rm UUU}}}^{{\rm Lys}} , {\rm tRNA}_{{\rm s^{2} {\rm UUG}}}^{{\rm Gln }} \;{\rm and}\;{\rm tRNA}_{{\rm s^{2} {\rm UUC}}}^{{\rm Glu}}}$ (Esberg et al. 2006; Chen et al. 2011; Tigano et al. 2015; Fernandez-Vazquez et al. 2013; Bauer et al. 2012; Nedialkova and Leidel 2015). Thus, we were interested in investigating whether the metabolic alterations in *elp3Δ* cells are suppressed by elevated levels of ${\text {tRNA}_{{\rm s^{2} {\rm UUU}}}^{{\rm Lys}} , {\rm tRNA}_{{\rm s^{2} {\rm UUG}}}^{{\rm Gln }} \;{\rm and}\;{\rm tRNA}_{{\rm s^{2} {\rm UUC}}}^{{\rm Glu}}}$. We compared the PLS-DA model of the *elp3Δ* strain that either contained an empty pRS425 high-copy vector (*elp3Δ*-h.c.-empty) or overexpressed ${\text {tRNA}_{{\rm s^{2} {\rm UUU}}}^{{\rm Lys}} , {\rm tRNA}_{{\rm s^{2} {\rm UUG}}}^{{\rm Gln }} \;{\rm and}\;{\rm tRNA}_{{\rm s^{2} {\rm UUC}}}^{{\rm Glu}}}$ (*elp3Δ*-h.c.-tKQE) with the PLS-DA model that compared the *elp3Δ*-l.c.-empty strain with the *elp3Δ*-l.c.-*ELP3* strain.

PLS-DA modelling of the comparison between the *elp3Δ*-h.c.-empty strain and the *elp3Δ*-h.c.-tKQE strain produced models with lower Q^2^-values than PLS-DA models of the comparison between the *elp3Δ*-l.c.-empty and *elp3Δ*-l.c.-*ELP3* strains (Figs. 3a–f, 4a–d, Online Resource 7). This result indicates that very few metabolic alterations are suppressed by overexpression of ${\text {tRNA}_{{\rm s^{2} {\rm UUU}}}^{{\rm Lys}} , {\rm tRNA}_{{\rm s^{2} {\rm UUG}}}^{{\rm Gln }} \;{\rm and}\;{\rm tRNA}_{{\rm s^{2} {\rm UUC}}}^{{\rm Glu}}}$ in the *elp3Δ* strain. However, the growth defect of the *elp3Δ* strain at 34 °C was partially suppressed by overexpression of the aforementioned tRNAs (data not shown).

At permissive (30 °C) growth we observed suppression of alteration of beta-alanine metabolism, and weak suppression of alterations of Glutamine, Tyrosine, Ornithine and Lysine metabolism from a comparison between metabolic patterns of identified metabolites from the *elp3Δ*-h.c.-empty and *elp3Δ*-h.c.-tKQE strains grown at 30 °C. We did not observe suppression of the altered metabolism of: Malic acid, Aspartic acid, Pyroglutamic acid, Alanine, Threonine, 2-Aminobutyric acid, 5,6-Dihydrouracil and *N*-Acetylglucosamine (Fig. 5a–e, Online Resource 2, 8).

Next, we investigated whether overexpression of ${\text {tRNA}_{{\rm s^{2} {\rm UUU}}}^{{\rm Lys}} , {\rm tRNA}_{{\rm s^{2} {\rm UUG}}}^{{\rm Gln }} \;{\rm and}\;{\rm tRNA}_{{\rm s^{2} {\rm UUC}}}^{{\rm Glu}}}$ in the *elp3Δ* strain led to a unique suppression pattern of metabolic alterations during growth at 34 °C. A comparison between the metabolite patterns of the *elp3Δ*-h.c.-empty and *elp3Δ*-h.c.-tKQE strains grown at 34 °C revealed that elevated levels of ${\text {tRNA}_{{\rm s^{2} {\rm UUU}}}^{{\rm Lys}} , {\rm tRNA}_{{\rm s^{2} {\rm UUG}}}^{{\rm Gln }} \;{\rm and}\;{\rm tRNA}_{{\rm s^{2} {\rm UUC}}}^{{\rm Glu}}}$ suppressed alterations in Lysine and Ornithine metabolism. However, changes in Beta-alanine and Glutamine metabolism were not suppressed in *elp3Δ*-h.c.-tKQE strains grown at 34 °C. Elevated levels of ${\text {tRNA}_{{\rm s^{2} {\rm UUU}}}^{{\rm Lys}} , {\rm tRNA}_{{\rm s^{2} {\rm UUG}}}^{{\rm Gln }} \;{\rm and}\;{\rm tRNA}_{{\rm s^{2} {\rm UUC}}}^{{\rm Glu}}}$ partially suppressed alterations in Lactic acid, Tyrosine, Alanine and Glutamic acid metabolism at 34 °C. Moreover, an analytical trend indicated that alterations in several other metabolites may be weakly suppressed (Fig. 5a–e, Online Resource 2, 9).

Several unidentified metabolites were suppressed in the *elp3Δ* strains at both 30 and 34 °C (Online Resource 10–11). Overall, our results indicate that suppression of certain metabolic alterations varies with elevated levels of ${\text {tRNA}_{{\rm s^{2} {\rm UUU}}}^{{\rm Lys}} , {\rm tRNA}_{{\rm s^{2} {\rm UUG}}}^{{\rm Gln }} \;{\rm and}\;{\rm tRNA}_{{\rm s^{2} {\rm UUC}}}^{{\rm Glu}}}$ in the *elp3Δ* strain depending on the growth temperature. However, Ornithine and Lysine are exceptions in the *elp3Δ* strain as alterations of these metabolites are partially suppressed with elevated levels of the three tRNAs at both 30 and 34 °C.

## Discussion

Mutations in genes encoding Elongator complex subunits have been linked to a multitude of phenotypes in *S. cerevisiae* (Otero et al. 1999; Wittschieben et al. 1999; Winkler et al. 2002; Rahl et al. 2005; Tigano et al. 2015; Nedialkova and Leidel 2015; Frohloff et al. 2001; Chen et al. 2011; Q. Li et al. 2009). Many investigations in Eukaryotes support a role for the complex in formation of the ncm^5^U, mcm^5^U and mcm^5^s^2^U wobble uridine nucleosides in tRNA [Reviewed in (Karlsborn et al. 2014b)]. Furthermore, phenotypes observed in yeast Elongator mutants can be suppressed by overexpression of various combinations of ${\text {tRNA}_{{\rm s^{2} {\rm UUU}}}^{{\rm Lys}} , {\rm tRNA}_{{\rm s^{2} {\rm UUG}}}^{{\rm Gln }} \;{\rm and}\;{\rm tRNA}_{{\rm s^{2} {\rm UUC}}}^{{\rm Glu}}}$ (Esberg et al. 2006; Chen et al. 2011; Tigano et al. 2015; Fernandez-Vazquez et al. 2013; Bauer et al. 2012; Nedialkova and Leidel 2015). This suppression has been ascribed to restoring translational efficiency of codons normally read by ${\text{tRNA}}_{{{\text{mcm}}^{{\text{5}}} {\text{s}}^{{\text{2}}} {\text{UUU}}}}^{{{\text{Lys}}}} {\text{,\; tRNA}}_{{{\text{mcm}}^{{\text{5}}} {\text{s}}^{{\text{2}}} {\text{UUG}}}}^{{{\text{Gln}}}} \;{\text{and}}\;{\text{tRNA}}_{{{\text{mcm}}^{{\text{5}}} {\text{s}}^{{\text{2}}} {\text{UUC}}}}^{{{\text{Glu}}}}$ by compensating the reduced codon-anticodon interaction of the hypomodified tRNAs with elevated levels of these three tRNA species (Esberg et al. 2006; Chen et al. 2011).

In this study, we used untargeted GC-TOF-MS based metabolomics to investigate the extent of metabolic alterations in an *elp3Δ* strain. We found that loss of the ncm^5^U, mcm^5^U and mcm^5^s^2^U wobble uridine nucleosides in tRNA resulted in an altered metabolism, and that only a subset of these alterations were suppressed by overexpression of ${\text {tRNA}_{{\rm s^{2} {\rm UUU}}}^{{\rm Lys}} , {\rm tRNA}_{{\rm s^{2} {\rm UUG}}}^{{\rm Gln }} \;{\rm and}\;{\rm tRNA}_{{\rm s^{2} {\rm UUC}}}^{{\rm Glu}}}$. This was surprising, as all phenotypes tested in yeast *elp3Δ* strains to date, except the tRNA modification defect, have been at least partially suppressed by elevated levels of various combinations of these three tRNAs (Esberg et al. 2006; Chen et al. 2011; Tigano et al. 2015; Fernandez-Vazquez et al. 2013; Bauer et al. 2012). Therefore, suppression of most of the metabolic alterations observed in an *elp3Δ* mutant may require elevated levels of additional tRNA species that normally have the ncm^5^U, mcm^5^U and mcm^5^s^2^U wobble uridine nucleosides.

It is possible that some metabolic alterations in the *elp3Δ* strain could be transient, or observed only when cells are exposed to certain stress conditions. Furthermore, metabolic alterations observed in the *elp3Δ* strain could, in part, be a global metabolic adaptation of the primary metabolic defects due to inefficient translation. If *elp3Δ* cells adapt to specific metabolic defects by reconfiguring global metabolism, the stress tolerance may be influenced, as the altered metabolism may cause the cell to be in an unfavourable state of metabolic homeostasis. Thus, overexpression of ${\text {tRNA}_{{\rm s^{2} {\rm UUU}}}^{{\rm Lys}} , {\rm tRNA}_{{\rm s^{2} {\rm UUG}}}^{{\rm Gln }} \;{\rm and}\;{\rm tRNA}_{{\rm s^{2} {\rm UUC}}}^{{\rm Glu}}}$ in the *elp3Δ* strain could suppress certain primary metabolic defects and alter the metabolic homeostasis into a more favourable state, resulting in a cellular metabolism better equipped to handle sudden changes in cell physiology due to stress exposure. To differentiate between primary and secondary metabolic defects in the *elp3Δ* strain, an instantaneous elimination of the ncm^5^U, mcm^5^U and mcm^5^s^2^U tRNA modifications is required. However, there are no known enzymes that specifically remove these modifications. Moreover, the long half-life of tRNAs means that a rapid depletion of Elongator would not generate an immediate loss of the modifications, but instead a gradual depletion of modified tRNA species during cellular growth.

Ribosomal profiling studies of Elongator mutants have shown increased ribosome pausing when lysine-AAA and glutamine-CAA codons are in the ribosomal A-site (Nedialkova and Leidel 2015). Increased ribosome pausing in an *elp6Δ* mutant can be alleviated by overexpression of ${\text {tRNA}_{{\rm s^{2} {\rm UUU}}}^{{\rm Lys}} , {\rm tRNA}_{{\rm s^{2} {\rm UUG}}}^{{\rm Gln }} \;{\rm and}\;{\rm tRNA}_{{\rm s^{2} {\rm UUC}}}^{{\rm Glu}}}$ which lack the mcm^5^- side chain (Nedialkova and Leidel 2015). These results indicate that global translation efficiency is affected by loss of these side chains in tRNA, and further, that defective translation due to increased ribosome pausing is suppressed by overexpression of the aforementioned tRNA species. Nonetheless, even if ribosomal pausing occurs on AAA and CAA codons, it is possible that only a few mRNAs have translation defects which result in altered protein expression and likely an altered metabolism.

## Concluding remarks

Overall, our metabolic profiling data shows that *elp3Δ* strains have widespread metabolic alterations. These metabolic alterations can be restored by complementation of the *elp3Δ* strains with the wild type *ELP3*, whereas only a few metabolic alterations are suppressed by overexpression of ${\text {tRNA}_{{\rm s^{2} {\rm UUU}}}^{{\rm Lys}} , {\rm tRNA}_{{\rm s^{2} {\rm UUG}}}^{{\rm Gln }} \;{\rm and}\;{\rm tRNA}_{{\rm s^{2} {\rm UUC}}}^{{\rm Glu}}}$. Our metabolic profiling also revealed unidentified metabolites which are altered in the *elp3Δ* strain. In the future, more comprehensive databases over yeast metabolites could allow identification of these metabolites, making it possible for our data set to be of valuable use in future studies of the Elongator complex in *Saccharomyces cerevisiae*.

## Electronic supplementary material

Below is the link to the electronic supplementary material.

Supplementary material 1 (PDF 130 kb)

Supplementary material 2 (PDF 263 kb)

Supplementary material 3 (PDF 261 kb)

Supplementary material 4 (PDF 322 kb)

Supplementary material 5 (PDF 215 kb)

Supplementary material 6 (PDF 225 kb)

Supplementary material 7 (PDF 131 kb)

Supplementary material 8 (PDF 266 kb)

Supplementary material 9 (PDF 328 kb)

Supplementary material 10 (PDF 217 kb)

Supplementary material 11 (PDF 230 kb)

Supplementary material 12 (XLSX 148 kb)

Supplementary material 13 (TXT 85 kb)
