# Supplementary material for: Loss of ncm5 and mcm5 wobble uridine side chains results in an altered metabolic profile
Source: Metabolomics. 2016 Sep 27;12(12):177. doi: 10.1007/s11306-016-1120-8 (PMC5037161; doi:10.1007/s11306-016-1120-8)
Supplement: Supplementary file 1 — Supplementary material 1 (PDF 130 kb) [file 11306_2016_1120_MOESM1_ESM.pdf]

Loss of ncm^5 and mcm^5 wobble uridine side chains results in an altered metabolic profile

Tony Karlsborn[1], A K M Firoj Mahmud[1†], Hasan Tükenmez[1†] and Anders S. Byström[1,*]
1) Department of Molecular Biology, Umeå University, 901 87 Umeå, Sweden
† These authors contributed equally
* Corresponding author, Phone (+46)-90-785 67 64; Fax (+46)-90-77 26 30
E-mail address, Anders.Bystrom@molbiol.umu.se
Metabolomics-Springer

Online Resource 1. Strains and plasmids used in this study.

Strain	Genotype	Source
**UMY4238**	MAT a *leu2Δ0*	This study
**UMY4239**	MAT a *leu2Δ0 elp3::kanMX4*	This study
**UMY2016/ UMY2026**	MAT a/α *ura3Δ0/URA3 leu2Δ0/LEU2*	This study
**Plasmid**	**Genotype**	**Source**
**ABY1653**	*pRS425-tRNAGlnUUG-tRNALysUUU-tRNAGluUUC*	(Lu et al. 2005)
**ABY525**	*pRS425*	(Christianson et al. 1992)
**ABY1514**	*pRS315-ELP3*	(Huang et al. 2005)
**ABY348**	*pRS315*	(Sikorski and Hieter 1989)
