# Supplementary material for: Loss of ncm5 and mcm5 wobble uridine side chains results in an altered metabolic profile
Source: Metabolomics. 2016 Sep 27;12(12):177. doi: 10.1007/s11306-016-1120-8 (PMC5037161; doi:10.1007/s11306-016-1120-8)
Supplement: Supplementary file 2 — Supplementary material 2 (PDF 263 kb) [file 11306_2016_1120_MOESM2_ESM.pdf]

Loss of ncm5 and mcm5 wobble uridine side chains results in an altered metabolic profile

Tony Karlsborn[1], A K M Firoj Mahmud[1†], Hasan Tükenmez[1†] and Anders S. Byström[1,*]

1) Department of Molecular Biology, Umeå University, 901 87 Umeå, Sweden

† These authors contributed equally

* Corresponding author, Phone (+46)-90-785 67 64; Fax (+46)-90-77 26 30

E-mail address, Anders.Bystrom@molbiol.umu.se

Metabolomics-Springer

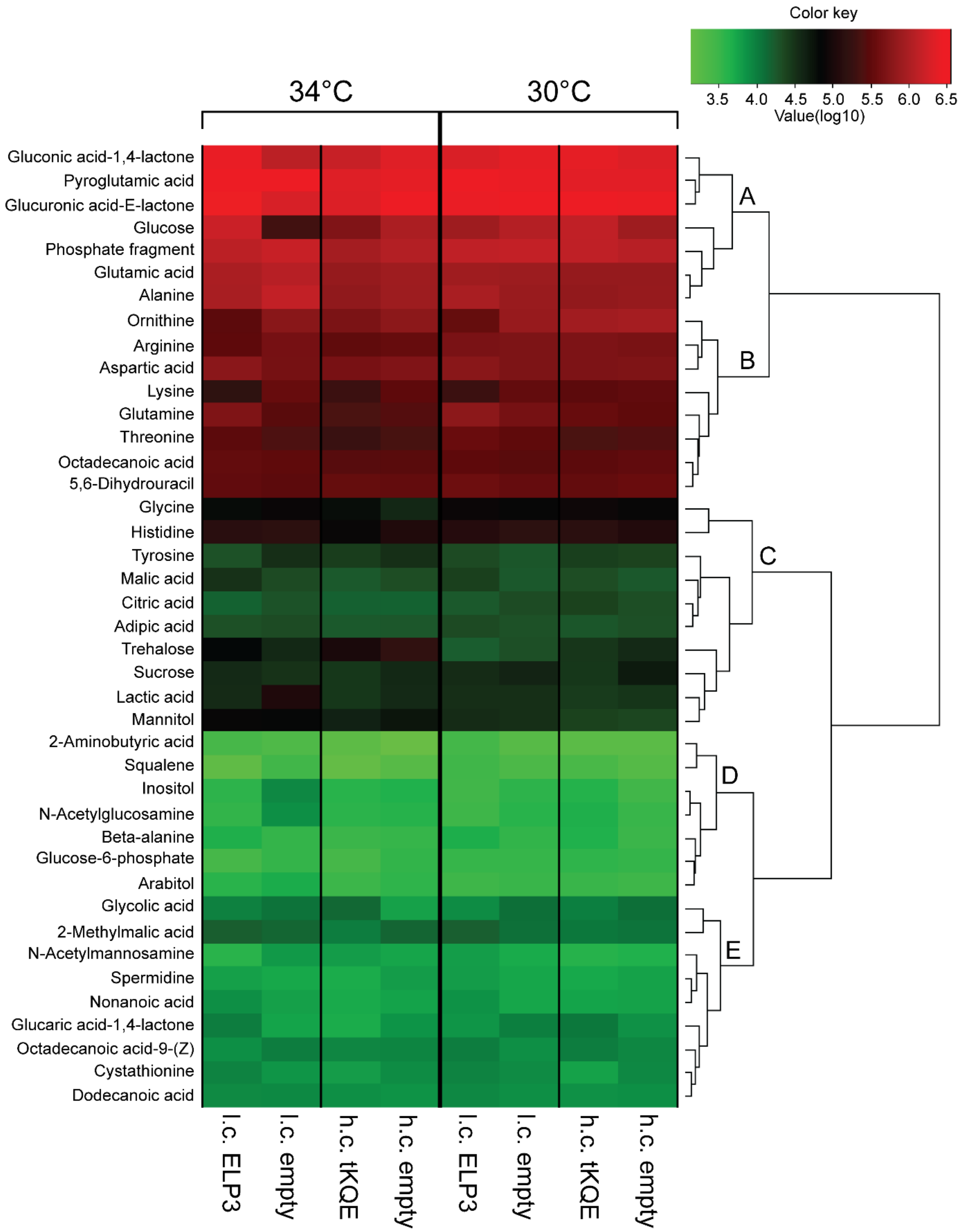

Online Resource 2. Hierarchal clustering of metabolites from strains containing indicated plasmids. The UMY4239 *elp3Δ* strains contained either: an empty pRS315 low copy vector (l.c. empty); a pRS315 vector carrying the wild-type *ELP3* gene (l.c. ELP3); an empty pRS425 high copy vector (h.c. empty); or a pRS425 high copy vector carrying the tRNA genes *tK(UUU)*, *tQ(UUG)* and *tE(UUC)* (h.c. tKQE). These yeast strains were grown logarithmically

to ~0.5 OD_{600} at 30°C or 34°C and harvested. Metabolites were extracted and then quantified using GC-TOF-MS. Hierarchical clusters are labeled from A to E based on metabolite abundance. Red signifies metabolite enrichment and green signifies metabolite reduction.
