# Supplementary material for: Loss of ncm5 and mcm5 wobble uridine side chains results in an altered metabolic profile
Source: Metabolomics. 2016 Sep 27;12(12):177. doi: 10.1007/s11306-016-1120-8 (PMC5037161; doi:10.1007/s11306-016-1120-8)
Supplement: Supplementary file 4 — Supplementary material 4 (PDF 322 kb) [file 11306_2016_1120_MOESM4_ESM.pdf]

Loss of ncm^5 and mcm^5 wobble uridine side chains results in an altered metabolic profile

Tony Karlsborn[1], A K M Firoj Mahmud[1†], Hasan Tükenmez[1†] and Anders S. Byström[1,*]
1) Department of Molecular Biology, Umeå University, 901 87 Umeå, Sweden
† These authors contributed equally
* Corresponding author, Phone (+46)-90-785 67 64; Fax (+46)-90-77 26 30
E-mail address, Anders.Bystrom@molbiol.umu.se
Metabolomics-Springer

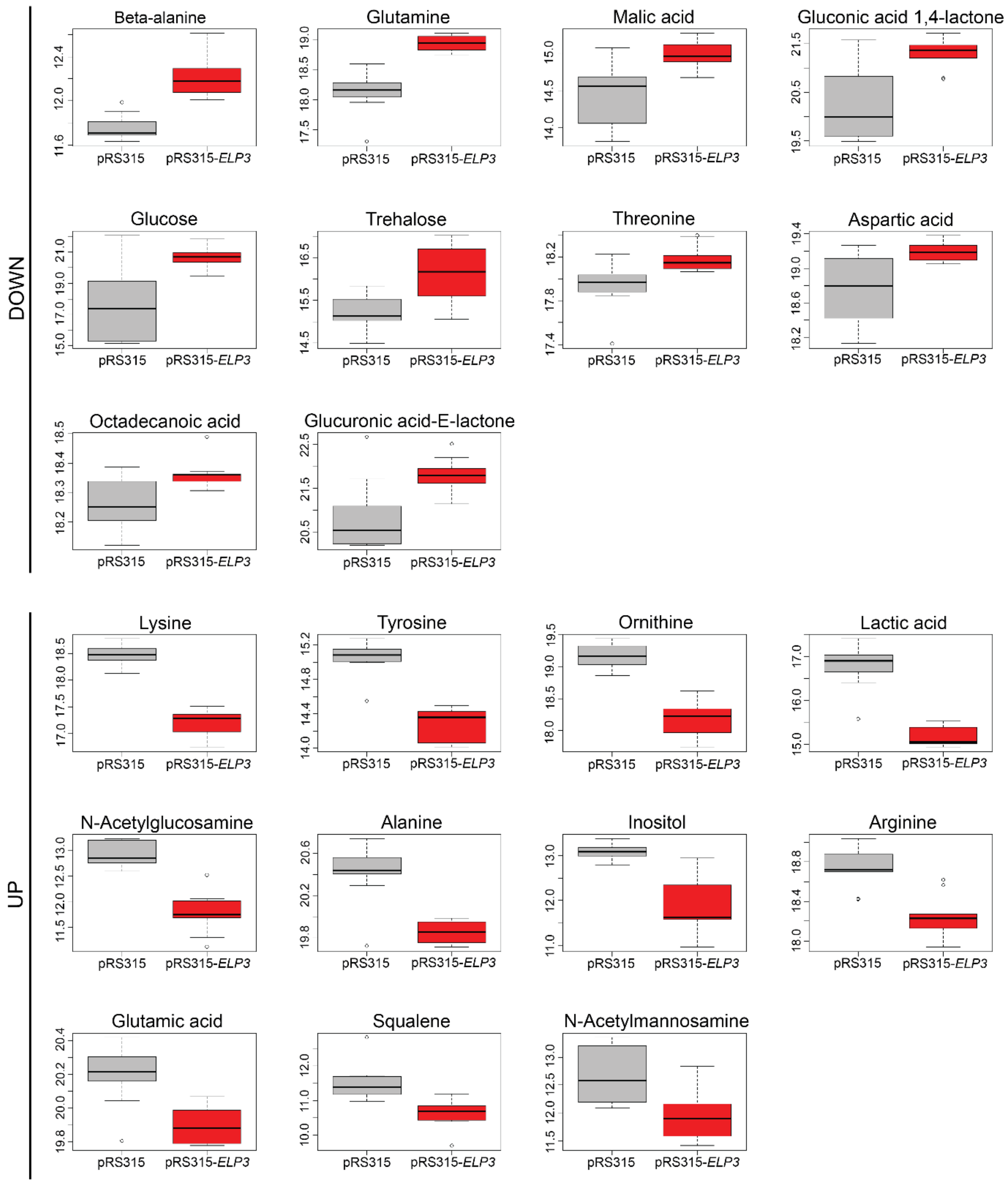

Online Resource 4. Levels of identified metabolites with a VIP score above 1 in the PLS-DA comparing the *elp3Δ*-l.c.-empty and *elp3Δ*-l.c.-ELP3 strains grown at 34°C. The *elp3Δ* strain containing either an empty low copy pRS315 vector or a pRS315 vector carrying the wild type *ELP3* gene was grown logarithmically to an OD_{600} of ~0.5 and harvested (see material and methods). Metabolites were extracted and then quantified using GC-TOF-MS. Values obtained were normalized and log2-transformed, and metabolic alterations were analyzed using PLS-DA. Metabolites shown have a VIP score >1. Metabolites were classified as either increasing (UP) or decreasing (DOWN) in the *elp3Δ* strain containing an empty low copy pRS315 vector when compared to the *elp3Δ* strain carrying the wild-type *ELP3* gene. Boxplots were generated using R software with the Y-axis displaying the relative intensity in log2-scale.
