# Supplementary material for: Loss of ncm5 and mcm5 wobble uridine side chains results in an altered metabolic profile
Source: Metabolomics. 2016 Sep 27;12(12):177. doi: 10.1007/s11306-016-1120-8 (PMC5037161; doi:10.1007/s11306-016-1120-8)
Supplement: Supplementary file 7 — Supplementary material 7 (PDF 131 kb) [file 11306_2016_1120_MOESM7_ESM.pdf]

Loss of ncm^5 and mcm^5 wobble uridine side chains results in an altered metabolic profile

Tony Karlsborn[1], A K M Firoj Mahmud[1†], Hasan Tükenmez[1†] and Anders S. Byström[1,*]
1) Department of Molecular Biology, Umeå University, 901 87 Umeå, Sweden
† These authors contributed equally
* Corresponding author, Phone (+46)-90-785 67 64; Fax (+46)-90-77 26 30
E-mail address, Anders.Bystrom@molbiol.umu.se
Metabolomics-Springer

Online Resource 7. Summary of the PLS-DA models used in the study.

Culturing Temp.	Strain	Obs.	Tot. Obs.	Comp.	Eigenv.	R2Y	Q2	CV-ANOVA
30°C	***elp3Δ*-l.c.-empty**	9	18	3	5.18	0.87	0.8	**3,38E-05**
30°C	***elp3Δ*-l.c.-ELP3**	9						
30°C	***elp3Δ*-h.c.-empty**	9	18	1	2.48	0.803	0.35	**0,0394747**
30°C	***elp3Δ*-h.c.-tKQE**	9						
30°C	***elp3Δ*-h.c.-empty**	9	18	3	6.22	0.765	0.643	**2,26E-05**
30°C	**WT-h.c.-empty**	9						
34°C	***elp3Δ*-l.c.-empty**	9	18	2	5.82	0.951	0.903	**2,67E-07**
34°C	***elp3Δ*-l.c.-ELP3**	9						
34°C	***elp3Δ*-h.c.-empty**	9	17*	4	7.39	0.498	0.383	**0,0479456**
34°C	***elp3Δ*-h.c.-tKQE**	8*						

Obs. Indicates the number of observations from each strain used in the PLS-DA model (three biological replicates with three technical replicates for each biological replicate).

***** One technical replicate was defined as an outlier in the PLS-DA model and was therefore excluded.

Tot. Obs. Indicates the total number of observations used in the PLS-DA model.

Comp. The number of components in the PLS-DA model.

Eigenv. Eigenvalue describes the length of the vector making up the first component of the PLS-DA model.

R2Y Fraction of Y variation modeled in the first component of the PLS-DA model.

Q2 Cross-validation of the R2-value for the first component in the PLS-DA model.

CV-ANOVA P-value from the CV-ANOVA significance test of the PLS-DA model. The 95% statistical significance was set to $P < 0.05$ and the 99.9% statistical significance was set to $P < 0.001$.
