# Supplementary material for: Loss of ncm5 and mcm5 wobble uridine side chains results in an altered metabolic profile
Source: Metabolomics. 2016 Sep 27;12(12):177. doi: 10.1007/s11306-016-1120-8 (PMC5037161; doi:10.1007/s11306-016-1120-8)
Supplement: Supplementary file 9 — Supplementary material 9 (PDF 328 kb) [file 11306_2016_1120_MOESM9_ESM.pdf]

Loss of ncm[5] and mcm[5] wobble uridine side chains results in an altered metabolic profile

Tony Karlsborn[1], A K M Firoj Mahmud[1†], Hasan Tükenmez[1†] and Anders S. Byström[1,*]
1) Department of Molecular Biology, Umeå University, 901 87 Umeå, Sweden
† These authors contributed equally
* Corresponding author, Phone (+46)-90-785 67 64; Fax (+46)-90-77 26 30
E-mail address, Anders.Bystrom@molbiol.umu.se
Metabolomics-Springer

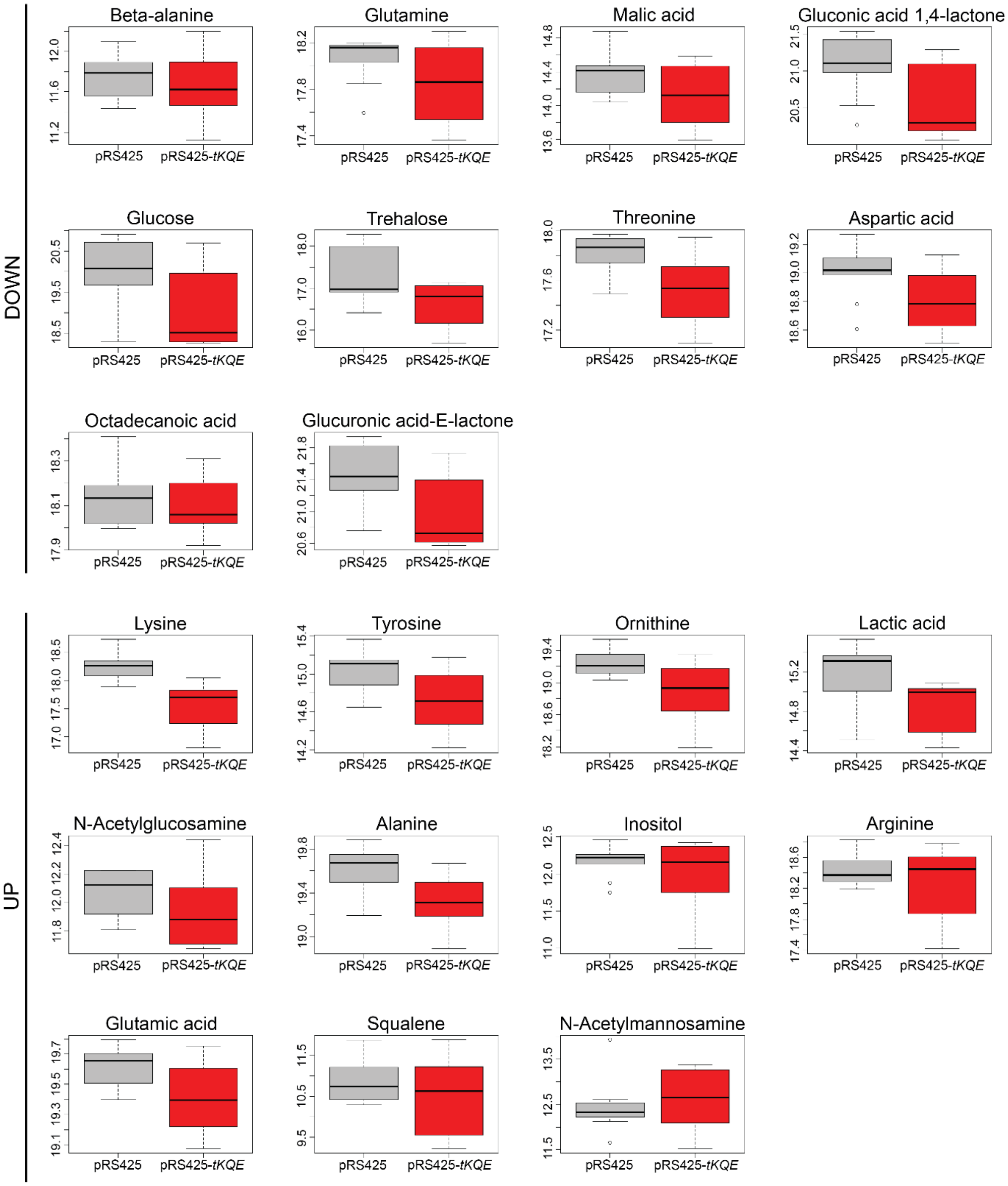

Online Resource 9. Suppression analysis comparing the *elp3Δ*-h.c.-empty and *elp3Δ*-h.c.-tKQE strains grown at 34°C. The *elp3Δ* strain containing either an empty high copy pRS425 vector or a pRS425 high copy vector carrying the tRNA genes *tK(UUU)*, *tQ(UUG)* and *tE(UUC)* (pRS425-tKQE) was grown logarithmically to an OD_{600} of ~0.5 and harvested (see material and methods). Metabolites were extracted and then quantified using GC-TOF-MS. Values obtained were normalized and log2-transformed, and metabolic alterations were analyzed using PLS-DA. Metabolites shown have a VIP score >1 in the PLS-DA model comparing the *elp3Δ*-l.c.-empty and *elp3Δ*-l.c.-ELP3 strains grown at 34°C. Metabolites were classified as either UP (increased) or DOWN (decreased) in the *elp3Δ*-l.c.-empty strain when compared with the *elp3Δ*-l.c.-ELP3 strain. Boxplots were generated using R software with the Y-axis displaying the relative intensity in log2-scale.
